# Supplementary material for: Using Decision Science for Monitoring Threatened Western Snowy Plovers to Inform Recovery
Source: Animals (Basel). 2021 Feb 22;11(2):569. doi: 10.3390/ani11020569 (PMC7926560; doi:10.3390/ani11020569)
Supplement: Supplementary file 1 [file animals-11-00569-s001.zip › Supplemental Appendix 1 Recovery Criteria.pdf]

Using Decision Science for Monitoring Threatened Western Snowy Plovers to Inform Recovery

Bruce G. Marcot, James E. Lyons, Daniel C. Elbert, and Laura Todd

Supplemental Appendix 1. Criteria for delisting the Western Snowy Plover from the Threatened species list. See USFWS (2007:142-147) for details.

The Pacific coast population of the western snowy plover will be considered for delisting when the following criteria have been met.

Criterion 1. Monitoring shows that an average of 3,000 breeding adults distributed among 6 recovery units have been maintained for a minimum of 10 years.

> 1a. A program is developed and implemented to monitor the western snowy plover breeding population and wintering locations to determine whether recovery unit subpopulation criteria are being achieved.
>
> 1b. A program is developed and implemented to monitor site-specific threats and monitoring results are used to refine site-specific management actions.
>
> 1c. Management activities that are necessary to ameliorate threats and achieve increases in reproductive success, survival, and overall population size are incorporated into participation and management plans developed and implemented under Criterion 3.
>
> 1d. Research actions are completed and incorporated into management and participation plans and into monitoring plans.

Criterion 2. A yearly average productivity of at least one fledged chick per male has been maintained in each recovery unit in the last 5 years prior to delisting.

Criterion 3. Mechanisms have been developed and are in place to assure long-term protection and management of breeding, wintering, and migration areas to maintain the subpopulation sizes and average productivity specified in Criteria 1 and 2.

> 3a. Working groups for each of the six recovery units are established.
>
> 3b. A participation plan for each recovery unit working group has been developed and implemented.
>
> 3c. Management plans for all Federal and State lands identified as pertinent to recovery of the species have been developed and implemented.
>
> 3d. Mechanisms to protect and manage western snowy plover breeding and wintering sites are in place for all areas owned or managed by local governments or private landowners.
>
> 3e. Public information and education programs are developed and implemented.
