# Supplementary material for: Using Decision Science for Monitoring Threatened Western Snowy Plovers to Inform Recovery
Source: Animals (Basel). 2021 Feb 22;11(2):569. doi: 10.3390/ani11020569 (PMC7926560; doi:10.3390/ani11020569)
Supplement: Supplementary file 1 [file animals-11-00569-s001.zip › Supplemental Appendix 2 Constructed Scales.pdf]

Using Decision Science for Monitoring Threatened Western Snowy Plovers to Inform Recovery

Bruce G. Marcot, James E. Lyons, Daniel C. Elbert, and Laura Todd

Supplementary Appendix 2. Performance measures and constructed scales assigned to the monitoring objectives.

Performance measures and constructed scales assigned to the monitoring objectives (Table 1). Accuracy was used as a performance measure for estimated population size, estimated annual survival, and estimated nest success rate. These constructed scales were used to evaluate the expected performance of various monitoring strategies.

**Performance Measure**	**Constructed scale: Score and Description of score**
Accuracy: bias and precision	5 = Very high (low bias + high precision)<br>4 = High (medium/high bias + high precision)<br>3 = Medium (low bias + medium precision)<br>2 = Medium low (medium/high bias + medium precision)<br>1 = Low (any level of bias + low precision)
Effectiveness: identifying causes of nest failures	5 = Substantially better than the historical average (> 10% reduction in nest failures attributed to "unknown cause")<br>4 = Better than the historical average (5% to 10% reduction in nest failures attributed to "unknown cause")<br>3 = No different than the historical average (0% to 5% reduction or increase in nest failures attributed to "unknown cause")<br>2 = Worse than the historical average (5% to 10% increase in nest failures attributed to "unknown cause")<br>1 = Substantially worse than the historical average (> 10% increase in nest failures attributed to "unknown cause")
Effectiveness: apportioning sources of nest predation	5 = Substantially better than the historical average (> 10% reduction in predation events attributed to "unknown predator")<br>4 = Better than the historical average (5% to 10% reduction in predation events attributed to "unknown predator")<br>3 = No different than the historical average (0% to 5% reduction or increase in predation events attributed to "unknown predator")<br>2 = Worse than the historical average (5% to 10% increase in predation events attributed to "unknown predator ")<br>1 = Substantially worse than the historical average (> 10% increase in predation events attributed to "unknown predator")
Information transfer: timeliness	5 = Information is reported daily<br>4 = Information is reported every other day<br>3 = Information is reported on intervals of one week or less<br>2 = Information is reported at bi-weekly intervals<br>1 = Information is reported on monthly intervals
Information transfer: availability	2 = Written report (information is distributed universally in writing and is formatted for record maintenance)<br>1 = Verbal report (information is distributed verbally to selected individuals and requires additional formatting for record maintenance)
