# Supplementary material for: Using Decision Science for Monitoring Threatened Western Snowy Plovers to Inform Recovery
Source: Animals (Basel). 2021 Feb 22;11(2):569. doi: 10.3390/ani11020569 (PMC7926560; doi:10.3390/ani11020569)
Supplement: Supplementary file 1 [file animals-11-00569-s001.zip › Supplemental Appendix 3 Sampling Strategies.pdf]

Using Decision Science for Monitoring Threatened Western Snowy Plovers to Inform Recovery

Bruce G. Marcot, James E. Lyons, Daniel C. Elbert, and Laura Todd

SAMPLING STRATEGIES Arranged by Objective to Facilitate Scoring

1. MAXIMIZE ACCURACY OF ESTIMATED ADULT POPULATION SIZE

Strategy A. Partially Marked Population (> 50%)

i. Multiple surveys (i.e., at least three) that measure the abundance of breeding adults at each site within a Recovery Unit will be conducted during an appropriate time interval that minimizes movement among sites and Recovery Units.
   1. The first survey will be conducted following the Breeding Window Survey protocol (Elliot-Smith and Haig 2007) explicitly.
   2. Subsequent surveys will be conducted based on the Breeding Window Survey protocol (Elliot-Smith and Haig 2007), but may be modified to include reading of band combinations.
   3. N-mixture models will be used during data analyses, to correct counts.

Strategy B. Varied Population Sizes

i. At all occupied, historically occupied, and unoccupied sites with potentially suitable habitat within the Recovery Unit, a survey will be conducted following the Breeding Window Survey (Elliot-Smith and Haig 2007).

ii. At sites within the Recovery Unit where populations of breeding adults are greater than 25 individuals:
   1. A minimum of two subsequent surveys will be conducted based on the Breeding Window Survey protocol (Elliot-Smith and Haig 2007), but may be modified to include reading of band combinations.

iii. At sites within the Recovery Unit where population of breeding adults are less than or equal to 25 individuals, a Banded Breeder Adjusted with Nest Ownership approach will be implemented:
   1. A count of banded and unbanded breeding adult males and females will be estimated by focusing on those birds that are found associated with a nest.
      a. Banded male and female adults identified on one or more nests in a breeding season within the Recovery Unit will be tallied to determine the total number of banded breeding adult males and females. Unbanded adults that are subsequently banded during the current breeding season are included in this estimate.
      b. The number of unbanded breeding adult males is the sum of unbanded males associated with broods at the end of the breeding season from each site within the Recovery Unit. This number may be augmented if a

higher number of simultaneous nests associated with an unbanded male are observed during the breeding season.

c. The number of unbanded breeding adult females is equal to the highest number of simultaneously active nests associated with an unbanded female during the breeding season.

Strategy C. Variable Plover Densities and Management Needs

i. Multiple surveys (i.e., at least three) that measure the abundance of breeding adults at each site within a Recovery Unit will be conducted during early May-June as feasible prior to most/all broods that minimizes movement among sites and Recovery Units.
   1. One of the surveys will be conducted following the Breeding Window Survey protocol (Elliot-Smith and Haig 2007) explicitly.
   2. Other surveys will be conducted with the normal N-mixture protocol.
   3. Estimates would be derived from N-mixture model analyses.

Strategy D. Minimal I Marked Population

i. Multiple surveys (i.e., at least three) that measure the abundance of breeding adults at each site within a Recovery Unit will be conducted during an appropriate time interval that minimizes movement among sites and Recovery Units.
   1. The first survey will be conducted following the Breeding Window Survey protocol (Elliot-Smith and Haig 2007) explicitly.
   2. Subsequent surveys will be conducted based on the Breeding Window Survey protocol (Elliot-Smith and Haig 2007), but may be modified to include reading of band combinations.
   3. N-mixture models will be used during data analyses, to correct counts.

Strategy E. Minimal II Effort/Resources

i. For Recovery Units with small population sizes:
   1. At all occupied, historically occupied, and unoccupied sites with potentially suitable habitat within the Recovery Unit, one survey will be conducted following the Breeding Window Survey (Elliot-Smith and Haig 2007) to measure the abundance of breeding adults.

ii. For Recovery Units with large populations sizes:
   1. Multiple surveys (i.e., at least three) that measure the abundance of breeding adults at each site within a Recovery Unit will be conducted during an appropriate time interval that minimizes movement among sites and Recovery Units.
      a. The first survey will be conducted following the Breeding Window Survey protocol (Elliot-Smith and Haig 2007) explicitly.

b. Subsequent surveys will be conducted based on the Breeding Window Survey protocol (Elliot-Smith and Haig 2007), but may be modified to include reading of band combinations.
c. N-mixture models will be used during data analyses, to correct counts.

Strategy F. Marked Individuals

i. Monitors will implement a Banded Breeder Adjusted with Counts approach to measure the abundance of breeding adults.
   1. Monitors will visit each nesting site within the Recovery Unit at least once every 10 days during May, June, and the first week of July.
   2. During these visits, presence of banded adults and counts the number of unbanded adults at each site are recorded.
   3. A minimum number of breeding adults is estimated by summing the number of unbanded adults observed during the 10-day interval with the cumulative tally of banded adults that were present at least once during the survey period.

Strategy G. Marked Population (b)

i. Monitors will implement a Peak Count Nests/Broods approach to measure the abundance of breeding adults.
   1. Monitors will survey each nesting site within the Recovery Unit throughout the breeding season.
   2. During each survey, the number of active nests (i.e., at least one egg is present in the nest bowl) and the number of chicks (and broods, if possible) are counted.
   3. At the end of the breeding season, the number of breeding adults is determined by:
      a. First, multiplying a correction factor (e.g., a ratio derived from previous measures of the number of males to females) to the number of nests observed during the peak nesting period (i.e., the one-week period during the breeding season when the most nests are active simultaneously), and then,
      b. Second, adding on the number of males that were observed associated with active broods during the period of peak nesting.

ii. Monitors will implement a Banded Breeder Adjusted with Counts approach to measure the abundance of breeding adults.
   1. Monitors will visit each nesting site within the Recovery Unit at least once every 10 days during May, June, and the first week of July.
   2. During these surveys, presence of banded adults and counts the number of unbanded adults at each site are recorded.

3. A minimum number of breeding adults is estimated by summing the number of unbanded adults observed during the 10-day interval with the cumulative tally of banded adults that were present at least once during the survey period.

Strategy H. Mostly Marked Population

i. Monitors will implement a Banded Breeder Adjusted with Counts approach to measure the abundance of breeding adults.
   1. Monitors will visit each nesting site within the Recovery Unit at least once every 10 days during May, June, and the first week of July in order to minimize movement among sites and Recovery Units.
   2. During these surveys, presence of banded adults and counts the number of unbanded adults at each site are recorded.
   3. A minimum number of breeding adults is estimated by summing the number of unbanded adults observed during the 10-day interval with the cumulative tally of banded adults that were present at least once during the survey period.

Strategy I.Nest Focused

i. Monitors will implement a Peak Count Nests/Broods approach to measure the abundance of breeding adults.
   1. Monitors will survey each nesting site within the Recovery Unit once per week throughout the breeding season.
   2. During each survey, the number of active nests (i.e., at least one egg is present in the nest bowl) and the number of chicks (and broods, if possible) are counted.
   3. At the end of the breeding season, the number of breeding adults is determined by:
      a. First, multiplying a correction factor (e.g., a ratio derived from historical measures of the number of males to females) to the number of nests observed during the peak nesting period (i.e., the one-week period during the breeding season when the most nests are active simultaneously), and then,
      b. Second, adding on the number of males that were observed associated with active broods during the period of peak nesting.

2. <u>MAXIMIZE ACCURACY OF ESTIMATED FLEDGLING PRODUCTIVITY</u>

Strategy A. Partially Marked Population (> 50%)

i. A sample of all chicks expected at each site within a Recovery Unit will be banded. All sites. From begin of season to end. Create 10 day periods; band first 3 broods in each 10 d period at each site.

ii. Each banded chick will be monitored for at least 28 days following hatching in order to determine the fate of banded individuals.

iii. Fledgling productivity for all chicks within a Recovery Unit will be estimated by dividing the number of chicks that fledge from the sample by the number of broods

in the sample. This can be multiplied by the number of males (as estimated in Objective 1) in the population for an overall number of chicks fledged.

Strategy B. Varied Population Sizes

i. For Recovery Units where the breeding adult population is greater than or equal to 250 individuals:
   1. Each week during the breeding season, attempt to find all new nests initiated at each site. 75% of nests found each week will be randomly selected for each site. All chicks from the randomly selected nests will be banded.
   2. Each banded chick will be monitored for at least 28 days following hatching in order to determine the fate of banded individuals.
   3. Fledgling productivity for all chicks within a Recovery Unit will be estimated by dividing the number of chicks that fledge from the sample by the number of broods in the sample. This can be multiplied by the number of males (as estimated in Objective 1) in the population for an overall number of chicks fledged.

ii. For Recovery Units where the breeding adult population is less than 250 individuals:
   1. Attempt to find all nests at each site and band all chicks within the study area.
   2. Each banded chick will be monitored for at least 28 days following hatching in order to determine the fate of banded individuals.
   3. Fledgling productivity for all chicks within a Recovery Unit will be estimated based on the proportion of banded chicks that are observed to have fledged during that breeding season.

Strategy C. Variable Plover Densities and Management Needs

i. A sample of all chicks stratified across all sites within the Recovery Unit and across the breeding season will be banded. Chicks at some sites may not be banded, if chick fate can be determined without individually marking chicks.
   1. Banding frequency will be reduced by limiting banding efforts to weekdays.

ii. Each banded chick will be monitored for at least 28 days following hatching to determine the fate of banded individuals.

iii. Fledgling productivity would be calculated or estimated for each site with the Recovery Unit, based on the monitoring methods for the site (in particular whether banding is employed) for all chicks will be estimated based on the proportion of banded chicks that are observed to have fledged during that breeding season.

Strategy D. Minimal I Marked Population

i. The goal of this strategy is to estimate fledgling survival rates in Recovery Units (or strata within Recovery Units) where lambda is less than 1.0 for several years. The goal is not to estimate number of chicks fledged per male.

ii. Approach:

1. Band a subset of chicks across all sites within the Recovery Unit (accounting for variation in space, time and sites).
2. Each banded chick will be monitored for at least 28 days following hatching in order to determine the fate of banded individuals.
3. Fledgling productivity for all chicks within a Recovery Unit will be estimated based on the proportion of banded chicks that are observed to have fledged during that breeding season.

iii. When the Recovery Unit rate of population growth (i.e. lambda) is greater than or equal to 1.0:
   1. There is no further action is needed. A lambda consistently or on average greater than 1.0 assumes that fledgling productivity needs are satisfactory.

Strategy E. Minimal II Effort/Resources

i. When the Recovery Unit breeding adult population growth rate (i.e., lambda) is less than 1.0 monitors will:
   1. Band a sample of chicks across all sites within the Recovery Unit will be banded.
   2. Each banded chick will be monitored for at least 28 days following hatching in order to determine the fate of banded individuals.
   3. Fledgling productivity for all chicks within a Recovery Unit will be estimated based the number of chicks fledged, the number of males tending sample broods, and the number of breeding males in the larger population.

ii. When the Recovery Unit rate of population growth (i.e. lambda) is greater than or equal to 1.0:
   1. There is no further action is needed. A lambda of greater than 1.0 assumes that fledgling productivity needs are satisfactory.

Strategy F. Marked Individuals

i. Monitors will band all chicks and nesting adult males and females at a subset of dedicated sites (approximately 25% of the total number of breeding sites within a Recovery Unit).

ii. Criteria for selection of dedicated sites within Recovery Units will include (1) north to south geographic representation, (2) public ownership, and (3) long-term protection and management measures for plovers have been established and are implemented.

iii. Each banded chick will be monitored for at least 28 days following hatching in order to determine the fate of banded individuals.

iv. The number of chicks per male that fledge at these dedicated sites during the breeding season will be reported as the annual productivity metric for the Recovery Unit.

Strategy G. Marked Population (b)

i. Monitors will attempt to band all chicks at all sites within a Recovery Unit.
ii. Each banded chick will be monitored for at least 28 days following hatching in order to determine the fate of banded individuals.
iii. Fledgling productivity for all chicks within a Recovery Unit will be estimated based on the proportion of banded chicks that successfully fledge divided by the number of males known to have nested.

Strategy H. Mostly Marked Population

i. A subset of all chicks (determined through power-analysis) will be stratified across all sites within the Recovery Unit and across the breeding season will be banded.
ii. Each banded chick will be monitored for at least 28 days following hatching in order to determine the fate of banded individuals.
iii. Fledgling productivity for all chicks within a Recovery Unit will be estimated based on the proportion of banded chicks that are observed to have fledged during that breeding season.

Strategy I.Nest Focused

i. Monitors will follow nests at all sites within a Recovery Unit until nest fate can be determined.
ii. An estimated number of fledglings for the Recovery Unit will be calculated by multiplying an average fledging rate (based on historical data) to the number of chicks observed to hatch.
iii. Fledgling productivity will be estimated by dividing the Recovery Unit estimate of fledglings by the Recovery Unit estimate of breeding males (banded and unbanded as determined by the Peak Count Nests/Broods method in Objective 1).

3. <u>MAXIMIZE ACCURACY OF ESTIMATED ANNUAL SURVIVAL OF ADULTS AND JUVENILES</u>

Strategy A. Partially Marked Population (> 50%)

i. Repeated counts (at least 3, within the peak breeding season to minimize effects of movement) of unmarked individuals at all sites within a Recovery Unit (or among Recovery Units) will be conducted in conjunction with mark-recapture techniques at some sites within a Recovery Unit(s).
ii. Count and mark-recapture data can be joined in an integrated population model for more robust estimates of adult and juvenile survival than is possible individually (Schaub and Abadi 2011, J. of Ornith. 152:227–237; Tempel et al. 2014, Ecol. Mod. 289:86–95).

Strategy B. Varied Population Sizes

i. Periodic mark-resight surveys are conducted at sites within the Recovery Unit that have individually banded birds.
ii. Mark-resight analyses are conducted to calculate annual survival of banded adults and juveniles.

Strategy C. Variable Plover Densities and Management Needs

i. Use observations of marked individuals from nest monitoring and other fieldwork to run mark-recapture analyses at sites at which banding is occurring.
ii. Overwinter survival of adults and juveniles will be estimated annually based on overwinter return rate of banded individuals.

Strategy D. Minimal I Marked Population

i. Fledgling survival for all chicks within a Recovery Unit will be estimated based on this sampling strategy. Use the overall lambda derived from the repeated counts of unmarked individuals within a Recovery Unit.
ii. Annual survival of adults and juveniles will be estimated using a closed population N-mixture model.

Strategy E. Minimal II Effort/Resources

i. Do not attempt to measure these response variables, since they are not recovery criteria.

Strategy F. Marked Individuals

i. Mark-resight surveys will be conducted at a subset of sites encompassing the dedicated banding sites and sites containing suitable habitat up to 15 km away from dedicated banding sites.
ii. Monitors will conduct a minimum of 5 mark-resight surveys throughout the breeding season. Each survey will be conducted at the beginning of each month in order to ensure that surveys are done at consistent times of year and to evenly space surveys, which will facilitate modelling of any seasonal effects.
iii. Surveys will be completed within 3 days of initiation (i.e., the duration of each survey will be < 10% of the interval length between surveys), in order to ensure that resight surveys are effectively instantaneous in comparison with the intervals between survey sessions.
iv. To maximize detection, surveys should be conducted during good weather and high visibility. On sunny days, visibility is best early in the morning or in the evening; visibility may be good at any hour on an overcast day. Rainy, foggy, or excessively windy conditions (15 mph or greater) are not suitable for surveying, however a slight drizzle or strong breeze (5-10 mph) is acceptable.
v. Annual survival analyses will be conducted using standard techniques (e.g., program MARK).

Strategy G. Marked Population (b)

i. Monitors will conduct mark-resight surveys at all sites within a Recovery Unit.

ii. Annual survival of adults and juveniles will be estimated based on overwinter return rate of banded individuals. Only banded individuals present in year *n* are considered for return in year *n+1*.

Strategy H. Mostly Marked Population

i. Monitors will conduct mark-resight surveys at all sites within a Recovery Unit.

ii. An index of annual survival of adults and juveniles will be estimated based on overwinter return rate of banded individuals. Only banded individuals present in year *n* are considered for return in year *n+1*.

Strategy I.Nest Focused

i. Monitors will coordinate among Recovery Units and conduct repeated occupancy surveys throughout the Pacific coast range during an appropriate time interval that minimizes movement among sites and Recovery Units.

ii. Survivorship of all adults and juveniles can be estimated using dynamic N-occupancy models (following Rossman et al. 2016, Ecology 97:3300–3307) after:

1. 10 years with a minimum of 25 sites across the range.
2. 5 years with a minimum of 75 sites across the range.

4. <u>MAXIMIZE UNDERSTANDING OF NEST FATE</u>

Strategy A. Partially Marked Population (> 50%)

iii. A sample of all nests across all sites within the Recovery Unit and across the breeding season will be monitored until nest fate can be determined.

iv. Nest cameras will be installed at each site based on spatial and temporal distribution of nests. Fate at all other sampled nests will be determined every three to seven days.

v. Nest fate will be determined using evidence (i.e. camera footage) that is recorded at sampled nests with nest cameras, or by evidence (i.e., animal sign, tracks, etc.) that is observed at sampled nests during physical nest checks.

Strategy B. Varied Population Sizes

i. When the Recovery Unit rate of population growth (i.e. lambda) is greater than or equal to 1.0:

1. At sites where the number of breeding adults is less than or equal to 25 individuals, all nests are monitored until nest fate can be determined.

2. At sites where the number of breeding adults is greater than 25 individuals, 75% of nests in the randomized sample (see Objective 2 methods) are monitored across the breeding season until nest fate can be determined.
3. Nest cameras will be installed on at least 30% of simultaneously active nests, which have been selected for monitoring. All other nests will be physically examined at least every three days.
4. Nest fate will be determined using evidence (i.e. camera footage) that is recorded at nests with nest cameras, or by evidence (i.e., animal sign, tracks, etc.) that is observed at nests during physical nest checks.

ii. When the Recovery Unit rate of population growth (i.e., lambda) is less than 1.0 in two consecutive years, or in three out of five years:

1. All nests at all sites are monitored until nest fate can be determined.
2. Nest cameras will be installed on at least 30% of simultaneously active nests. All other nests will be physically examined at least every three days.
3. Nest fate will be determined using evidence (i.e. camera footage) that is recorded at sampled nests with nest cameras, or by evidence (i.e., animal sign, tracks, etc.) that is observed at sampled nests during physical nest checks.

Strategy C. Variable Plover Densities and Management Needs

i. At managed areas, all nests at all sites within the Recovery Unit throughout the breeding season are monitored until nest fate can be determined.

1. At primary managed areas (relatively large number of breeders and land manager investment in management), nest checks are conducted at least every three days to physically examine the nest site and its contents.
2. At second tier managed areas (i.e., adjacent to key managed areas), nest checks may be conducted at intervals greater than every three days (but at least weekly if possible), to physically examine the nest site and its contents.
3. If there is a good deal of nest loss, nest cameras will be installed at a portion of all nests.
4. Nest fate will be determined using evidence (i.e., animal sign, tracks, etc.) that is observed at nests during physical nest checks. Camera footage recorded at nests with nest cameras will be used to determine nest fate for nests with nest cameras.

ii. At sites where there is minimal or no management occurring, nest searches and checks are made less regularly (e.g. once a week if resources permit, but less frequently if not).

Strategy D. Minimal I Marked Population

i. An adaptive sampling scheme based on environmental factors will be implemented to monitor a representative sample of nests until nest fate can be determined.

ii. This adaptive sampling scheme will only be implemented at sites within the Recovery Unit when the Recovery Unit (or strata within the Recovery Unit) population growth rate (i.e., lambda) is less than 1.0 for several consecutive years.
iii. Nest cameras will be installed at some of the sampled nests (accounting for temporal and spatial variation). All other sampled nests will be physically examined at least every three days. The relative proportion of nests monitored by cameras vs. physical nest checks by people will be assessed on a site-by-site basis depending on potential risk of cameras, human activity, etc.
iv. Nest fate will be determined using evidence (i.e. camera footage) that is recorded at sampled nests with nest cameras, or by evidence (i.e., animal sign, tracks, etc.) that is observed at sampled nests during physical nest checks.

Strategy E. Minimal II Effort/Resources

i. When the Recovery Unit rate of population growth (i.e. lambda) is less than 1.0, monitors will:
    1. Sample a portion of all nests across all sites within the Recovery Unit and across the breeding season, and monitor nests until nest fate can be determined.
    2. Install nest cameras at some of the sampled nests. All other sampled nests will be physically examined at least every three days. The relative proportion of camera vs. human monitored nests will be determined on a site-by-site assessment.
    3. Determine nest fate using evidence (i.e. camera footage) that is recorded at sampled nests with nest cameras, or using evidence (i.e., animal sign, tracks, etc.) that is observed at sampled nests during physical nest checks.

ii. When the Recovery Unit rate of population growth (i.e. lambda) is consistently greater than or equal to 1.0, monitors will:
    1. Not attempt to measure response variables associated with nest fate, since there are no recovery criteria directly associated with nest fate.

Strategy F. Marked Individuals

i. Monitors will visit each nesting site within the Recovery Unit at least once every 10 days during the breeding season.
ii. Any nest found on or prior to June 30 will be monitored until nest fate can be determined. Any nest found after June 30 will not be monitored.
iii. Nest cameras will be installed on 50% of all active nests at each nesting site. All remaining nests will be monitored by physically examining nests at least once every three days.
iv. Nest fate will be determined using evidence (i.e. camera footage) that is recorded at nests with nest cameras, and by using physical evidence (e.g., egg remains, animal sign, tracks, etc.) that is observed at nests during nest checks.

Strategy G. Marked Population (b)

i. All nests at all sites within the Recovery Unit will be monitored throughout the breeding season until nest fate can be determined.
   1. All nests will be physically examined at least every three days.
   2. Nest fate will be determined using evidence (i.e., animal sign, tracks, etc.) that is observed at nests during physical nest checks.

Strategy H. Mostly Marked Population

i. All nests at all sites within the Recovery Unit will be monitored throughout the breeding season until nest fate can be determined.
   1. Nest cameras will be installed at some of the nests. All other nests will be physically examined at least every three days.
   2. Nest fate will be determined using evidence (i.e. camera footage) that is recorded at sampled nests with nest cameras, or by evidence (i.e., animal sign, tracks, etc.) that is observed at sampled nests during physical nest checks.

Strategy I.Nest Focused

i. All nests at all sites within the Recovery Unit will be monitored throughout the breeding season until nest fate can be determined.
   1. Nest cameras will be installed on at least 50% of simultaneously active nests. All other nests will be physically examined at least once per week.
   2. Nest fate will be determined using evidence (i.e. camera footage) that is recorded at sampled nests with nest cameras, or by evidence (i.e., animal sign, tracks, etc.) that is observed at sampled nests during physical nest checks.

5. MAXIMIZE INFORMATION AVAILABLE TO MANAGERS

Strategy A. Partially Marked Population (> 50%)

i. Monitors will disseminate information related to objectives 1-4 to land management agency staff during a Weekly preparation and distribution of monitoring reports throughout the snowy plover breeding season.

Strategy B. Varied Population Sizes

i. Monitors will prepare a written report and disseminate information related to objectives 1-4 for all sites within the Recovery Unit to land management agency staff on an annual basis.
   1. When the Recovery Unit rate of population growth (i.e. lambda) is greater than or equal to 1.0, monitors will also:
      a. Participate in ad-hoc conference calls initiated by the WSP Working Group Chair or Predator Subcommittee Chair and prepare ad-hoc monitoring reports as directed by the WSP Working Group or Predator

Subcommittee during the breeding season to disseminate information related to objectives 1-4 to land management agency staff.

2. When the Recovery Unit rate of population growth (i.e., lambda) is less than 1.0 in two consecutive years, or in three out of five years, monitors will also:
   a. Participate in ad-hoc conference calls a initiated by the WSP Working Group Chair or Predator Subcommittee Chair and prepare ad-hoc monitoring reports as directed by the WSP Working Group or Predator Subcommittee during the breeding season to disseminate information related to objectives 1-4 to land management agency staff.
   b. Prepare written reports and disseminate information related to objectives 1-4 to land management agency staff on a weekly basis throughout the snowy plover breeding season.

Strategy C. Variable Plover Densities and Management Needs

i. Monitors will prepare a written report and disseminate information related to objectives 1-4 for all sites within the Recovery Unit to land management agency staff on an annual basis.

ii. Monitors will participate in ad-hoc communication (conference calls or emails) with management staff to disseminate information related to objectives 1-4 during the breeding season.

iii. Monitors meet at least every other week, but also as needed, with land managers and predator control staff to assess needs and priorities for various sites and subsites.

Strategy D. Minimal I Marked Population

i. When the Recovery Unit rate of population growth (i.e. lambda) is consistently less than 1.0, monitors will:
   1. Prepare a written report and disseminate information related to objectives 1-4 for all sites within the Recovery Unit to land management agency staff on a weekly basis.

ii. When the Recovery Unit rate of population growth (i.e. lambda) is greater than or equal to 1.0, monitors will:
   1. Provide a quick assessment of population trends upon completion of the final adult survey of the season and a final report after the N-mixture analysis. Any management concerns observed during surveys will be conveyed upon completion of each survey.

Strategy E. Minimal II Effort/Resources

i. Monitors will participate in a monthly conference call with land management agency staff to disseminate information related to objectives 1-4 during the breeding season.

ii. Monitors will prepare a written report and disseminate information related to objectives 1-4 for all sites within the Recovery Unit to land management agency staff on a monthly basis.

Strategy F. Marked Individuals

i. Monitors will prepare a written report and disseminate information related to objectives 1-4 to land management agency staff on a weekly basis throughout the snowy plover breeding season and on an annual basis.
ii. Monitors will participate in ad-hoc conference calls as needed (but no more than once per week) during the breeding season to disseminate information related to objectives 1-4 to land management agency staff.

Strategy G. Marked Population (b)

i. Monitors will prepare a written report and disseminate information related to objectives 1-4 to land management agency staff on a weekly basis throughout the snowy plover breeding season and on an annual basis.
ii. Monitors will participate in ad-hoc conference calls as needed during the breeding season to disseminate information related to objectives 1-4 to land management agency staff.

Strategy H. Mostly Marked Population

i. Monitors will individually call/email land management agency staff as needed and at least weekly during the breeding season to disseminate information related to objectives 1-4.

Strategy I.Nest Focused

i. Monitors will prepare a written report and disseminate information related to objectives 1-4 to land management agency staff on a weekly basis throughout the snowy plover breeding season and on an annual basis.
ii. Monitors will participate in monthly conference call with land management agency staff as needed during the breeding season to disseminate information related to objectives 1-4.
