# Supplementary material for: Using Decision Science for Monitoring Threatened Western Snowy Plovers to Inform Recovery
Source: Animals (Basel). 2021 Feb 22;11(2):569. doi: 10.3390/ani11020569 (PMC7926560; doi:10.3390/ani11020569)
Supplement: Supplementary file 1 [file animals-11-00569-s001.zip › Supplemental Appendix 4 Form for Scoring.pdf]

Using Decision Science for Monitoring Threatened Western Snowy Plovers to Inform Recovery
Bruce G. Marcot, James E. Lyons, Daniel C. Elbert, and Laura Todd

Supplemental Appendix 4. Form for scoring performance measures of monitoring objectives under monitoring sampling strategies. Values follow the scoring scales in Supplemental Appendix 2, with examples of entries from one team member shown here.

		Sampling Strategy								
**Monitoring Objective**	**Performance Measure**	**A. Partially Marked Population (>50%)**	**B. Varied Population Sizes**	**C. Variable Plover Densities & Mgt Needs**	**D. Minimum I Marked Pop**	**E. Minimal II Effort / Resources**	**F. Marked Individuals**	**G. Marked Population**	**H. Mostly Marked Population**	**I. Nest Focused**
1. Maximize accuracy of estimated adult population size	Accuracy (bias and precision)	3	3	3	3	2	5	5	3	2
2. Maximize accuracy of estimated fledging productivity	Accuracy (bias and precision)	3	5	3	2	2	2	5	4	1
3. Maximize accuracy of estimated annual survival of adults and juveniles	Accuracy (bias and precision)	4	3	3	3	3	5	4	4	3
4.1 Maximize accuracy of estimated nest success	Accuracy (bias and precision)	2	4	3	2	2	3	5	5	5
4.2 Minimize percent failures attributed to unknown cause	Effectiveness when identifying causes of nest failure	3	4	3	3	3	2	3	5	4
4.3 Minimize % of predation events attributed to unidentified predators	Effectiveness when apportioning sources of nest predation	3	4	3	3	3	2	3	5	4
5.1 Maximize actionable information available to managers: Timeliness	Timeliness of Information	3	3	2	3	1	3	3	3	3
5.2 Maximize actionable information available to managers: Availability	Availability of Information	2	2	1	2	2	2	2	1	2

		Sampling Strategy								
***Instructions for Scoring Costs*:** FIRST, put 100 in the cell for the most expensive strategy. SECOND, fill in the other cells with a number between 1 and 100; ties are allowed. Provide scores relative to the most expensive strategy (100); for example, if a strategy would cost one-half as much as the most expensive strategy, enter 50 in the cell for the cost of that strategy.		**A. Partially Marked Population (>50%)**	**B. Varied Population Sizes**	**C. Variable Plover Densities & Mgt Needs**	**D. Minimum I Marked Pop**	**E. Minimal II Effort / Resources**	**F. Marked Individuals**	**G. Marked Population**	**H. Mostly Marked Population**	**I. Nest Focused**
6. Cost	Relative cost	70	90	80	65	65	80	100	95	80
