# Supplementary material for: Using Decision Science for Monitoring Threatened Western Snowy Plovers to Inform Recovery
Source: Animals (Basel). 2021 Feb 22;11(2):569. doi: 10.3390/ani11020569 (PMC7926560; doi:10.3390/ani11020569)
Supplement: Supplementary file 1 [file animals-11-00569-s001.zip › Supplemental Appendix 5 Rationale Provided by Team Members.pdf]

Using Decision Science for Monitoring Threatened Western Snowy Plovers to Inform Recovery

Bruce G. Marcot, James E. Lyons, Daniel C. Elbert, and Laura Todd

Supplemental Appendix 5. Rationale provided by the 8 Western Snowy Plover Technical Team members as documented for their Round 2 scoring of performance of each monitoring objective for each monitoring sampling strategy. Team members also provided their initial rationale for their Round 1 scoring, which they subsequently discussed in a guided discussion session, and were then provided the opportunity to retain or amend their rationale for Round 2 scoring. Only their final Round 2 recorded rationale is presented here.

See text Table 1 for monitoring objectives and their performance measures, and text Table 2 and Supplementary Appendix 3 for explanation of monitoring sampling strategies.

A. Rationale for scoring, for overall key evidence, uncertainties, and assumptions.

| **Monitoring Sampling Strategy** | **Rationale for your scoring of Performance Measures:**<br>**Key Strengths, Key Uncertainties, Key Assumptions** |
|---|---|
| A. Partially Marked Population (>50%) | Sample design in Objective 2 seemed like it would result in an under-representative sample, and was not really specified in other Objectives. |
| Varied Population Sizes | Assumed that N-mixture models were used to correct breeding adult counts, as mentioned in other alternatives for Objective 1.<br>In Recovery Unit 1, the population is above 250, so [this monitoring sampling strategy] is irrelevant to Oregon and would not be implemented. Due to the difficulties in determining nest ownership, I would say the bias is medium and because of uncertainty (because this depends on positively identifying adults at nests - a difficult task), then the precision is medium too.<br>Doubt many land managers would have time for a weekly conference call. |
| C. Variable Plover Densities and Management Needs | In Objective 2, approach 1 seemed like it would introduce more bias and result in lower precision than the 2nd approach. Score had to be averaged.<br>For Objective 2, I really don't know the bias or the precision of this method, so it is difficult for me to score it, so I just went with medium bias and medium precision. |
| D. Minimum I Marked Population | Strategy relies more on estimating response variables by deriving them from population growth rate rather than directly measuring the response variable, which could potentially result in less precision.<br>For Objective 2, it states that it does not measure fled/male, so it does not address the objective which is to measure fled/male, so I gave it a 1 but would haven't given it a 0 if possible because it doesn't meet the objective (not sure why it is being considered?); same for Objective 4 - if above lambda, this is not being measured, so how do you score something that is not being measured? |

| | |
|---|---|
| E. Minimal II Effort / Resources | Strategy relies more on estimating response variables by deriving them from population growth rate rather than directly measuring the response variable,  which could potentially result in less precision.<br>For Objective 2, again one of the strategies does not meet the objective, so not sure how this is being considered and scored.  There is a lot of bias in all these calculations, and I'm not convinced that anything other than low precision is possible as there will be no way to measure how accurate it is.  In general if this strategy does not meet a number of the objectives, which it does not, then why are we considering it? |
| F. Marked Individual | Assumed that only the maximum count (not all counts) of unbanded adults was used to estimate breeding adult population.<br>Nests initiated after 30 June not monitored, but it appears that chicks are banded and monitored all season. |
| G. Marked Population | Assumed that only the maximum count (not all counts) of unbanded adults was used to estimate breeding adult population. |
| H. Mostly Marked Population | Assumed that only the maximum count (not all counts) of unbanded adults was used to estimate breeding adult population.<br>Unclear what proportion of nests would have cameras installed. |
| I. Nest Focused | Focuses on nests and can inform all three spatial scales (rangewide, recovery unit and site-specific). |

B. Rationale for scoring by monitoring objective.

| **Monitoring Objective** | **Performance Measure** | **Rationale for your scoring of Strategies:<br>Key Strengths, Key Uncertainties, Key Assumptions** |
|---|---|---|
| 1. Maximize accuracy of estimated adult population size | Accuracy: bias and precision | Key strengths included consistency in using a methodology throughout the breeding season. Strategies C [Varied Population Sizes] and D [Variable Plover Densities and Management Needs] applied the Breeding Window Survey protocol to all survey periods, whereas other strategies allowed for modifications to the Breeding Window Survey protocol that could introduce additional sources of error, and therefore increase bias and reduce precision of the estimate). Strategies H [Marked Population] and J [Nest Focused] scored low, because of known sources of bias (a set correction factor to determine the ratio of males to females), and the potential of nest predation to cause the period of peak nesting to vary across the range (among recovery units or sites within recovery units) in such a way that could significantly reduce precision.<br>N-mixtures - I'm not convinced the precision is any better than medium.<br>For this objective, I felt that the strategies outlined were not as distinct as our measure categories and would have preferred to give score of 4.5 to my 5's, and 3.5 to my 3's. Also, I am still putting my faith in the n-Mixture approach, although I think it still deserves a critical evaluation with plovers at high densities.<br>N mixture - the accuracy is unknown but bias is very low - assume medium accuracy but it could be higher. For varied pop. Size, this is not a statistical estimate so bias and precision will be unknown (bias could be very high especially with "augmentation"). High counts have known biases. Strategy G [Marked Individuals] - not sure how the repeated counts will be used. Strategy H [Marked Population] - really worry about correction factors (they usually have an unknown variance). |

| | | |
|---|---|---|
| 2. Maximize accuracy of estimated fledging productivity | Accuracy: bias and precision | Strategies E [Minimum I Marked Population] and F [Minimal II Effort / Resources] were identical and relied on estimates of breeding adult population from Objective 1 (which scored lower, as explained above), or did not identify what proportion of chicks would be banded (and therefore were not scored any higher than strategy A, which accounted for how a subset of chicks would be sampled across space and time, but still scored low). Strategies C [Varied Population Sizes], H [Marked Population], and J [Nest Focused] scored high because it was clear that a large proportion, or all chicks would be banded across all sites within an RU (e.g., more replicates), which would result in a more precise estimate (precision being the more important of the two indices of the performance measure as noted in the goals and objectives document). Strategy D [Variable Plover Densities and Management Needs] had two possible approaches that were not equal in how I scored them (the first approach would only band and monitor 50% of breeding males - roughly 50% of broods?, while the second approach attempted to adaptively band and monitor as many broods as possible - likely many more than 50% of chicks/broods), so I averaged the scores of the two possible approaches.<br>For Strategy A [Partially Marked Population > 50%], bias depends on the size of the population, if pop high, then bias is high, if low, then bias less.<br>Minimum I/II- high precision where measured, but low precision where not measured - overall med precision, med bias - measurements biased towards the populations that are lambda<1; Strategies G [Marked Individuals] & I [Mostly Marked Population] seem to be the same.<br>Historic values really worry me - there is no reason to believe that the past will predict the future, especially as the population gets larger and there is more intraspecific interaction. |

| | | |
|---|---|---|
| 3. Maximize accuracy of estimated annual survival of adults and juveniles | Accuracy: bias and precision | Strategies C [Varied Population Sizes], H [Marked Population], and I [Mostly Marked Population] scored low because they estimated return rates rather than survival, and were less precise while still having similar amounts of bias to other strategies that scored higher (i.e., E [Minimum I Marked Population], F [Minimal II Effort / Resources], and G [Marked Individuals]). Strategies A [Partially Marked Population > 50%], D [Variable Plover Densities and Management Needs] and J [Nest Focused] scored higher because they incorporated methods to reduce known sources of bias (e.g., coverage error - integrating mark-recapture techniques with unmarked counts [Strategy A [Partially Marked Population > 50%]], non-response error - increasing the capture period [4-5 years in Strategy D [Variable Plover Densities and Management Needs] & J [Nest Focused]] or estimation error - integrating a simple and consistent sampling technique across the entire range [Strategy J [Nest Focused]]), while also incorporating survival analyses.<br>For Strategy G [Marked Individuals]: Depends on how "dedicated banding sites" are selected. If selected in a manner that they are not representative, then bias will be high. |
| 4. Maximize understanding of nest fate - 4.1 Maximize accuracy of estimated nest success | Accuracy: bias and precision | Key assumptions: (1) inclusion of nest cameras and greater frequency of nest checks (i.e. at least every 3 days) will reduce known sources of bias (coverage error, non-response error, response error, estimation error, and processing error) related to physical nest checks. (2) Alternatives that did not specify the proportion of nests that will be sampled were nevertheless assumed to include a sufficient number of replicates to accurately reflect the true population of nests.<br>For Strategy E [Minimum I Marked Population], I put "1" because if no nests are monitored, then it is a "1". If nest are monitored, it could be "3", but there is no way to score it with 2 numbers. Same for Strategy F [Minimal II Effort / Resources].<br>First tier and second tier approach worries me because of the obvious bias. Strategy G [Marked Individuals] - won't the value derive prior to June 30th will be biased in an unpredictable way? |

| | | |
|---|---|---|
| 4. Maximize understanding of nest fate - 4.2 Minimize percent failures attributed to unknown cause | Effectiveness: identifying causes of nest failures | I scored alternatives with nest checks every 3 days higher than alternatives with 7 day nest checks, since we are more likely to be able to identify cause of failure the more frequently we check. Alternatives with cameras scored higher.<br>Key assumption/strength: Increasing use of nest cameras would increase effectiveness of attributing causes of nest failure.<br>Since cameras are already a tool in our techniques for determining nest fate, I didn't think that there was much chance to substantially improve the rate of known cause of loss through any of these methods. I also think that cameras have their own set of logistic problems.<br>Still similar scores to 4c, but measures are appropriate; detectability of cause of failure and predator I.D. is similar. Most are at least a effective as current scheme, didn't see any that were better than current, some are less effective.<br>Strategy C [Varied Population Sizes] - the scores for this will change depending on which approach is used - e.g., all nests vs. 30%. |
| 4. Maximize understanding of nest fate - 4.3 Minimize % of predation events attributed to unidentified predators | Effectiveness: apportioning sources of nest predation | I scored alternatives with nest checks every 3 days higher than alternatives with 7 day nest checks, since we are more likely to be able to identify cause of failure the more frequently we check. Alternatives with cameras scored higher.<br>Key assumption/strength: Increasing use of nest cameras would increase effectiveness of attributing identification of predators.<br>See comments for 4.2.<br>Still similar scores to 4b, but measures are appropriate; detectability of cause of failure and predator I.D. is similar. Most are at least a effective as current scheme, didn't see any that were better than current, some are less effective. |

| | | |
|---|---|---|
| 5. Maximize actionable information available to managers | Information transfer: timeliness | For variable alternatives, I scored as if lambda was < 1.<br>Ad-hoc communication was difficult to score, but was assumed to occur more frequently than a more regularly scheduled reporting mechanism (e.g., weekly or bi-weekly report). In alternatives where this occurred, (i.e., Strategy D [Variable Plover Densities and Management Needs],Strategy H [Marked Population], and Strategy I [Mostly Marked Population]), the strategy was scored one level better than the regularly scheduled reporting mechanism. Strategy E [Minimum I Marked Population] was averaged between the two approaches described in that alternative.<br>For Strategy I [Mostly Marked Population], this is not scorable on this scale, because there are two totally different timelines.<br>I bumped up scores by 1 for each strategy that included ad-hoc or as needed communication to deal with urgent management issues. Otherwise we didn't need to score these as their score is obvious by the criteria and strategy description.<br>Strategy D [Variable Plover Densities and Management Needs] could get several scores because it is adaptive. This is true for most and make scoring using these criteria impossible. |
| 5. Maximize actionable information available to managers | Information transfer: availability | Key strength: Strategies that included universal and written communication scored a 2.<br>Strategy I [Mostly Marked Population] was the only strategy to score 1 on this alternative. Even though it included emails (i.e., written communication) in the description, the strategy indicated these communications would be distributed individually.<br>Is our scoring necessary for this objective, score is obvious by the strategy description?<br>Only scored for the in-season reports since all strategies would require an annual written report.<br>Strategy C [Varied Population Sizes] should get a 1 and a 2; Strategy D [Variable Plover Densities and Management Needs] should get a 1 and 2 - many provide a written and oral depending on the situation. Again, scoring is difficult using these criteria. |

C. Rationale for scoring of cost.

| Strategy | Rationale for your scoring of cost |
|---|---|
| A. Partially Marked Population (>50%) | Somewhat less expensive because a subset (though large subset) of broods are tracked. Nests are checked every 3-7 days (less expensive than every 3 days).<br>Scored high on Objective 3 but moderately for other Objectives.<br>All these strategies cost about the same to me because they all require a reasonable amount of bodies and time in the field, and the differences are fairly minor but do not really reduce the time spent in the field.<br>All season, all sites, sampling, chick banding, (no ad banding?) |
| B. Varied Population Sizes | Monitors a high proportion of nests and broods. Nest checks every 3 days.<br>Scored high on Objective 2, and moderately for other Objectives.<br>For cost purposes, I am assuming lambda < 1. All season, all sites, sampling and comprehensive monitoring, chick banding. Flexible strategy complicates cost estimation. |
| C. Variable Plover Densities and Management Needs | Monitor all nests and some broods. Nest checks every 3 days or 7 days depending on site. Some Mark/resight surveys.<br>Scored high on Objectives 3 and 4, and moderately for other objectives.<br>All season, all sites, sampling, chick and some adult banding. Flexible strategy complicates cost estimation. |
| D. Minimum I Marked Population | Strategy E [Minimum I Marked Population] and Strategy F [Minimal II Effort / Resources] are least expensive, repeated window surveys, only monitor a portion of broods when lambda <1. Nest checks every 3 days, but presumably a small portion of nests will be sampled.<br>Scored low on all Objectives.<br>Cannot determine cost because it is either the same as the above strategies, or it is substantially less because you aren't doing much of anything - I put in 50 just to score it.<br>For cost purposes, I am assuming lambda < 1. Mostly survey based, but a sample of chicks banded. |
| E. Minimal II Effort / Resources | Strategy E [Minimum I Marked Population] and Strategy F [Minimal II Effort / Resources] are least expensive, repeated window surveys, only monitor a portion of broods when lambda <1. Nest checks every 3 days, but presumably a small portion of nests will be sampled.<br>Scored low on all Objectives.<br>Same comment as above - are we measuring the cost based on a high or low lambda? I put in 50 just to score it.<br>For cost purposes, I am assuming lambda < 1. Mostly survey based, but a sample of chicks banded. |
| F. Marked Individual | Only 25% of nests monitored, 3 day nest checks. Relatively higher reporting burden. Mark/resight surveys.<br>Scored moderately high on all Objectives.<br>No monitoring of nests initiated after 30 June, comprehensive monitoring at dedicated sites, adult and chick banding. |

| | |
|---|---|
| G. Marked Population | Most expensive - attempts to monitor all nests/broods, checks nests every 3 days. No cameras. Mark/resight surveys.<br>Scored moderately high to high on all Objectives.<br>All season, all sites, comprehensive chick banding, assuming some adult banding. Flexible strategy complicates cost estimation. |
| H Mostly Marked Population | Attempts to monitor all nests/broods, nests without cameras checked every 3 days. Mark/resight surveys.<br>Scored high to very high on all Objectives.<br>All season, all sites, sampling, chick banding, assuming some adult banding. |
| I. Nest Focused | Nests without cameras will be checked weekly. Cost of nest cameras/maintenance.<br>Scored high on Objective 1 and really high on Objective 4 (nest success and predation), but lower on other objectives (e.g., no banding and limited survival survey work).<br>All season, all sites, fledglings not banded or monitored. |
