# Supplementary material for: Using Decision Science for Monitoring Threatened Western Snowy Plovers to Inform Recovery
Source: Animals (Basel). 2021 Feb 22;11(2):569. doi: 10.3390/ani11020569 (PMC7926560; doi:10.3390/ani11020569)
Supplement: Supplementary file 1 [file animals-11-00569-s001.zip › Supplemental Appendix 6 Parameters of Utility Functions.pdf]

Using Decision Science for Monitoring Threatened Western Snowy Plovers to Inform Recovery
Bruce G. Marcot, James E. Lyons, Daniel C. Elbert, and Laura Todd

Supplemental Appendix 6. Parameter values of the value-function model for each monitoring objective (text Table 1) scored by the Western Snowy Plover Technical Team for each monitoring sampling strategy (text Table 2). Minimum and maximum scores indicate the extremes for performance measures as scored by the Technical Team. Monotonicity indicates whether the value function is an increasing or decreasing function and rho is the exponential constant used in the value function (Kirkwood 1997).

**Monitoring Objective**	**Minimum score**	**Maximum score**	**Monotonicity**	**Rho**
1. Maximize accuracy of estimated adult population size	2.17	3.50	increasing	0.5
2. Maximize accuracy of estimated fledgling productivity	1.83	5.00	increasing	infinity
3. Maximize accuracy of estimated survival of adults and juveniles	2.67	4.00	increasing	infinity
4.1 Maximize understanding of nest fate: Maximize accuracy of estimated nest success	2.33	4.67	increasing	infinity
4.2 Maximize understanding of nest fate: Minimize percent nest failures with unknown cause	2.50	4.50	increasing	infinity
4.3 Maximize understanding of nest fate: Minimize percent of unidentified predators	2.50	4.50	increasing	infinity
5.1 Maximize information available to managers: Time, as required by the monitoring staff to prepare all the appropriate information	1.17	3.33	increasing	infinity
5.2 Maximize information available to managers: How the information is conveyed by the monitoring staff	1.33	2.00	increasing	infinity
6. Cost	48.50	98.83	decreasing	15
