# Supplementary material for: Using Decision Science for Monitoring Threatened Western Snowy Plovers to Inform Recovery
Source: Animals (Basel). 2021 Feb 22;11(2):569. doi: 10.3390/ani11020569 (PMC7926560; doi:10.3390/ani11020569)
Supplement: Supplementary file 1 [file animals-11-00569-s001.zip › Supplemental Appendix 7 Panelists.pdf]

Using Decision Science for Monitoring Threatened Western Snowy Plovers to Inform Recovery

Bruce G. Marcot, James E. Lyons, Daniel C. Elbert, and Laura Todd

Supplementary Appendix 7. Names and affiliations of the Western Snowy Plover Technical Team members and expert panelists providing information and scoring of monitoring performance of the alternative monitoring strategies. The collective expertise of members included analysis and modeling of wildlife populations, monitoring of Western Snowy Plover populations, shorebird ecology and behavior, and endangered species recovery.

Name	Affiliation	Title
Scott Pearson	Washington Department of Fish and Wildlife	Senior Researcher
Eleanor Gaines	Oregon Biodiversity and Information Center	Director and Zoologist
Dave Lauten	Oregon Biodiversity and Information Center	Faculty Research Assistant
Gary Page	Point Blue Conservation Science	Biologist
Lynne Stenzel	Point Blue Conservation Science	Senior Wetland Ecologist
Jim Watkins	U.S. Fish and Wildlife Service	Rangewide Species Lead Biologist for the western snowy plover
Daniel Elbert	U.S. Fish and Wildlife Service	Recovery Unit 1 (Washington and Oregon) Species Lead Biologist for the western snowy plover
Laura Todd	U.S. Fish and Wildlife Service	Field Supervisor
